# Coley's Fluid in Sarcoma

**Published:** 1902-05-31

**Authors:** 


### COLEY'S FLUID IN SARCOMA.

Little as we may be able to understand the action of Coley's mixed toxins in sarcoma, every case in which a malignant disease has been cured by their means is of extreme interest. Dr. Winberg[1] relates the case of a man, aged 40, who was struck over the right superior maxilla in February 1901. Two or three weeks later he had severe pain over the site of the injury, and in April a diagnosis of sarcoma was made. In May the upper jaw was excised. A large tumour was found involving the antrum and almost the entire upper jaw. The whole of the tumour could not be removed. There was also a mass the size of a hen's egg under the ear on the opposite side, and this was completely removed. The tumour of the jaw, which had been found by microscopical examination to be a sarcoma, soon began to grow again, and by August the patient had become extremely weak. He also had jaundice with dulness and tenderness in the hepatic region. The tumour extended from the right malar eminence to the bridge of the nose, with which it was even in height, and downwards to the edge of the maxilla. On removing a portion it was found to be "a large spindle-celled sarcoma." The disease rapidly progressed, the liver was enlarged, and the jaundice was profound. The pulse rose to 140–150 and was irregular and intermittent. Speech was difficult, and the odour from the disintegrating portion of the tumour was most offensive. The first injection was made on August 12th, 1901. The patient's condition grew worse, and on August 15th was so desperate that no treatment was given and it was thought best to abandon the use of the toxins. The patient, however, insisted on their being continued, which was done in gradually increasing doses. On August 17th there was suppression of urine, intense jaundice, the patient could not see, and could rarely make himself understood; the original tumour was the size of a man's fist and there was a swelling under the left jaw the size of a walnut. On August 19th, however, a slight improvement was seen and this soon became rapid and continuous. The toxins were kept up in large doses up to January 4th, 1902—in all 103 injections having been given. By September 4th he had increased 11 lbs. in weight and had a voracious appetite, and on January 13th, when Dr. Coley presented the patient before the New York Academy of Medicine, examination showed no trace of the tumour either in the neck, face, or jaw, nor was there anything abnormal in the abdomen. Commenting on this case Dr. Coley says that it fully demonstrates the correctness of the view which he has always maintained, that the curative action of the mixed toxins of erysipelas and bacillus prodigiosus as used by him is systemic as well as local, for in this case none of the injections were made into the tumour. The case, he truly says, is so extraordinary that it is most important that the diagnosis should be placed beyond question, and with this object, in addition to recording the opinion of several other observers by whom it was examined, he appends the detailed report of the microscopical examination made by Dr. William H. Welch, of Johns Hopkins University, who defines it as a "round-celled sarcoma of an accinous gland;" adding, "There is, perhaps, enough diversity in the cells to justify the designation mixed-cell sarcoma, but the round or oval cells greatly predominate. The tumour has the histological characteristics of malignancy." Clinically the occasional occurrence of such cases as the above is profoundly interesting, while pathologically it is puzzling to a degree.

[1] Med. Record, May 3.

